# Regulation of systemic energy homeostasis by serotonin in adipose tissues

**DOI:** 10.1038/ncomms7794

**Published:** 2015-04-13

**Authors:** Chang-Myung Oh, Jun Namkung, Younghoon Go, Ko Eun Shong, Kyuho Kim, Hyeongseok Kim, Bo-Yoon Park, Ho Won Lee, Yong Hyun Jeon, Junghan Song, Minho Shong, Vijay K. Yadav, Gerard Karsenty, Shingo Kajimura, In-Kyu Lee, Sangkyu Park, Hail Kim

**Affiliations:** 1 BioMedical Research Center (E7) 8104, Graduate School of Medical Science and Engineering, Korea Advanced Institute of Science and Technology, Daejeon 305-701, Korea; 2 Department of Biochemistry, Yonsei University Wonju College of Medicine, Wonju 220-701, Korea; 3 Department of Internal Medicine, Kyungpook National University Hospital, Kyungpook National University School of Medicine, Daegu 700-721, Korea; 4 BK21 Plus KNU Biomedical Convergence Program, Kyungpook National University Hospital, Kyungpook National University School of Medicine, Daegu 700-842, Korea; 5 Department of Nuclear Medicine, Kyungpook National University Hospital, Kyungpook National University School of Medicine, Daegu 700-721, Korea; 6 Leading-edge Research Center for Drug Discovery and Development for Diabetes and Metabolic Disease, Kyungpook National University Hospital, Kyungpook National University School of Medicine, Daegu 702-210, Korea; 7 Department of Laboratory Medicine, Seoul National University Bundang Hospital, Seoul National University College of Medicine, Seoul 463-707, Korea; 8 Research Center for Endocrine and Metabolic Diseases, Chungnam National University School of Medicine, Daejeon 301-721, Korea; 9 Wellcome Trust Sanger Institute, Cambridge CB10 1SA, UK; 10 Department of Genetics and Development, Columbia University Medical Center, New York, New York 10032, USA; 11 Diabetes Center, Hormone Research Institute and Department of Medicine, University of California San Francisco, San Francisco, California 94143, USA; 12 Department of Biochemistry, College of Medicine, Catholic Kwandong University, Gangneung 210-701, Korea

## Abstract

Central serotonin (5-HT) is an anorexigenic neurotransmitter in the brain. However, accumulating evidence suggests peripheral 5-HT may affect organismal energy homeostasis. Here we show 5-HT regulates white and brown adipose tissue function. Pharmacological inhibition of 5-HT synthesis leads to inhibition of lipogenesis in epididymal white adipose tissue (WAT), induction of browning in inguinal WAT and activation of adaptive thermogenesis in brown adipose tissue (BAT). Mice with inducible *Tph1* KO in adipose tissues exhibit a similar phenotype as mice in which 5-HT synthesis is inhibited pharmacologically, suggesting 5-HT has localized effects on adipose tissues. In addition, *Htr3a* KO mice exhibit increased energy expenditure and reduced weight gain when fed a high-fat diet. Treatment with an Htr2a antagonist reduces lipid accumulation in 3T3-L1 adipocytes. These data suggest important roles for adipocyte-derived 5-HT in controlling energy homeostasis.

## Abstract (web summary)

The neurotransmitter serotonin has both central and peripheral effects. Here, the authors show that adipocyte-derived serotonin regulates organismal energy homeostasis in mice by acting on adipocyte serotonin receptors on fat cells, which regulates lipolysis and thermogenesis in white and brown fat tissue.

## Graphical abstract

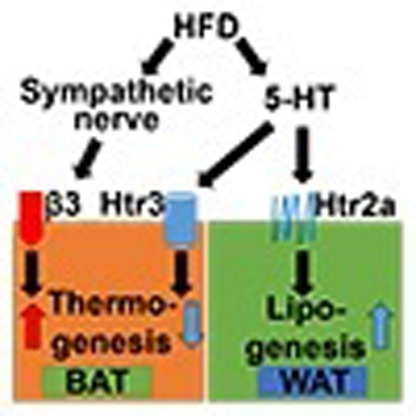

5-Hydroxytryptamine (5-HT, serotonin) is a monoamine that modulates central and peripheral functions. It is primarily found in the gastrointestinal tract, platelets, pineal gland and the central nervous system. 5-HT is synthesized from the essential amino acid tryptophan by the sequential actions of tryptophan hydroxylase (Tph) and aromatic amino acid decarboxylase. Hydroxylation of tryptophan is the initial and rate-limiting step in the synthesis of 5-HT. There are two isoforms of Tph: Tph1 and Tph2. Tph1 is primarily expressed in peripheral tissues, whereas Tph2 is exclusively expressed in neuronal tissues including the central nervous system and enteric neurons^1^. 5-HT commonly acts locally in neural and paracrine circuits, and it has a variable function depending on the tissue^2^. The action of released 5-HT is terminated by uptake into cells through 5-HT transporter (SERT)^3^.

As 5-HT cannot cross the blood–brain barrier, central and peripheral 5-HT systems are functionally separated. Almost 90% of body 5-HT is synthesized peripherally in the gastrointestinal tract and stored in platelets. Small amount of 5-HT is also present in other peripheral tissues^4^. Once released, 5-HT exerts its biological action by binding to 5-HT receptor (Htr). More than 14 Htrs have been identified and they are G-protein-coupled receptor except for Htr3, which is a ligand-gated cation channel.

Central 5-HT functions as an anorexigenic neurotransmitter by activating the Htr2c in the brain^5^,^6^,^7^,^8^. Direct intracranial injection of p-chlorophenylalanine (PCPA), a Tph inhibitor, into the ventricle induced marked hyperphagia and obesity^9^. However, body weight was reduced in *Tph1* and *Tph2* knockout (KO) mice^10^. Mice with a SERT-null mutation (*Slc6a4* KO) are expected to be slim due to the increased 5-HT activity, but these mice exhibit an obese phenotype^11^. The enhancement of 5-HT activity using a selective SERT inhibitor was associated with weight loss, but the effect was transient and restoration occurred during maintenance period^12^. These discordant results suggest that peripheral 5-HT might have opposite functions to central 5-HT in the regulation of energy homeostasis.

Here we show that 5-HT has a functional role in adipose tissues. We inhibited 5-HT synthesis in mice genetically by inducing *Tph1* KO in adipose tissue and pharmacologically by administrating the systemic Tph inhibitor PCPA^13^ and the peripheral Tph inhibitor LP-533401 (ref. 14). Under high-fat diet (HFD) condition, the inhibition of 5-HT synthesis reduced body weight gain, improved glucose tolerance, increased thermogenic activity in brown adipose tissue (BAT) and decreased lipogenesis in white adipose tissue (WAT). We also show that 5-HT inhibited thermogenesis through Htr3 in BAT and increased lipogenesis through Htr2a in WAT. Our data indicate that adipocyte-derived 5-HT plays important roles in controlling energy homeostasis and might be a therapeutic target for obesity and metabolic disease.

## Results

### Reduced weight gain by inhibiting 5-HT synthesis

**Figure 1:**
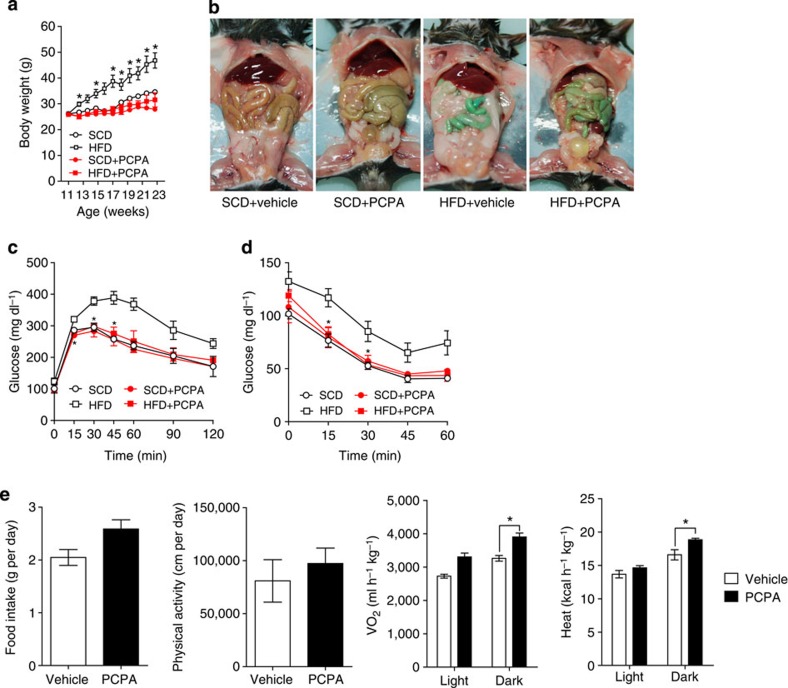
PCPA protects against diet-induced obesity. (**a**) Growth curves of vehicle- or PCPA-treated mice fed an SCD or HFD. *n*=4 mice per group. *P<0.05 versus HFD+PCPA by Student's *t*-test. (**b**) Gross images of vehicle- or PCPA-treated mice after 10 weeks of HFD feeding. (**c**) Intraperitoneal glucose tolerance test (IPGTT) after fasting for 16 h. *n*=3 mice per group. *P<0.05 versus HFD by Student's *t*-test. (**d**) Intraperitoneal insulin tolerance test (IPITT) after 4 h fasting. *n*=3 mice per group. *P<0.05 versus HFD by Student's *t*-test. (**e**) The metabolic rates of vehicle- or PCPA-treated mice after 6 weeks of HFD feeding. The metabolic parameters were measured using an 8-chamber Oxymax system. Mice were acclimatized to cages for 24 h and data were collected for an additional 48 h. *n*=4 mice per group. *P<0.05 versus vehicle by Student's *t*-test. All data are presented as the mean±s.e.

We hypothesized that if peripheral 5-HT has opposite effects to central 5-HT in the regulation of body weight, long-term systemic inhibition of 5-HT synthesis may reduce body weight or the degree of weight gain by an HFD. In this regard, mice were fed an HFD and administered PCPA by intraperitoneal injection for 12 weeks from 11 weeks of age. PCPA-treated mice ate more food than control mice during the first week of HFD, but their food intake became comparable to control mice from the second week throughout the HFD period. These changes of eating patterns matched well with previous reports^9^. As a result of the systemic inhibition of 5-HT synthesis, PCPA-treated mice exhibited decreased body weight gain on an HFD (Fig. 1a) and their visceral fat mass was reduced (Fig. 1b), although they showed similar body weight on a standard chow diet (SCD).

The severe loss of visceral fat mass after 12 weeks of PCPA treatment concerned that massive destruction of adipose tissue might cause lipodystrophy. However, we could find small fat cells with normal structure in WAT (Supplementary Fig. 1a). PCPA treatment improved glucose tolerance and insulin sensitivity in the HFD-fed mice (Fig. 1c,d). The serum levels of total cholesterol, free fatty acid and leptin were decreased in accordance with the reduction of WAT in the PCPA-treated mice (Supplementary Fig. 1b). Intriguingly, serum adiponectin levels were also decreased in PCPA-treated mice (Supplementary Fig. 1b). As 5-HT is known to decrease adiponectin expression in 3T3-L1 adipocytes^15^ and adiponectin improves insulin sensitivity, PCPA treatment was expected to increase serum adiponectin levels, which the improved insulin sensitivity could be attribute to. Thus, the decreased serum adiponectin in PCPA-treated mice is more likely to be an indirect reflection of reduced fat mass rather than the direct downregulation of adiponectin expression by inhibiting 5-HT synthesis in adipose tissues.

To investigate the mechanism of reduced weight gain following systemic administration of PCPA, we analysed the food intake and energy expenditure using indirect calorimetry. Under HFD condition, PCPA-treated mice showed higher oxygen consumption and heat production than control mice that could not be attributed to changes in food intake or physical activity (Fig. 1e and Supplementary Fig. 1c). However, PCPA treatment did not affect the metabolic rates of mice fed an SCD (Supplementary Fig. 1d), suggesting the requirement of metabolic stress for the positive effect of PCPA on energy expenditure. These data suggested that peripheral 5-HT affected positively on body weight control in contrast to central 5-HT.

### Inhibition of 5-HT synthesis increased thermogenesis

**Figure 2:**
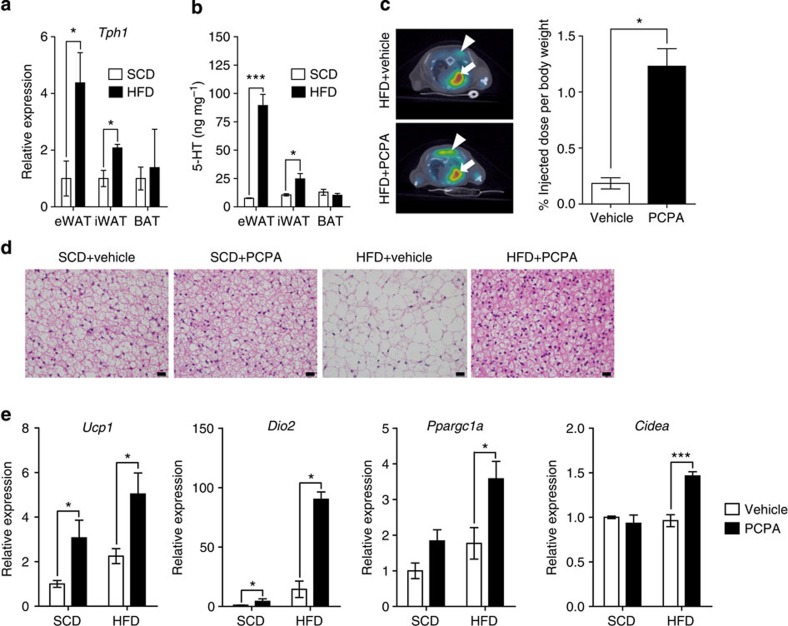
PCPA increased metabolic activity in BAT. (**a**) *Tph1* mRNA expression in adipose tissues was assessed by quantitative reverse transcriptase–PCR (qRT–PCR) after 2 weeks of HFD feeding. *n*=4 mice per group. *P<0.05 versus SCD by Student's *t*-test. (**b**) Tissue 5-HT levels were assessed by LC-MS after 2 weeks of HFD feeding. *n*=4 mice per group. *P<0.05 and ***P<0.001 versus SCD by Student's *t*-test. (**c**) Metabolic activity of BAT of vehicle- or PCPA-treated mice after 6 weeks of HFD feeding was assessed by PET-computed tomography (CT). Representative axial PET-CT images (left panel) and quantitative comparisons of ^18^fluorodeoxyglucose (^18^F-FDG) uptake in PET-CT images (right panel). BAT (triangle) and the heart (arrow) are highlighted. *P<0.05 versus vehicle by Student's *t*-test. (**d**) Haematoxylin and eosin (H&E) staining of BAT sections from vehicle- or PCPA-treated mice after 8 weeks of HFD feeding. Scale bar, 20 μm. (**e**) Expression of thermogenesis-associated genes in BAT, as assessed by qRT–PCR. *n*=5 per group. *P<0.05 and ***P<0.001 versus SCD by Student's *t*-test. All data are presented as the mean±s.e. LC–MS: liquid chromatography–mass spectrophotometry.

In peripheral tissues, 5-HT is mainly produced and secreted by enterochromaffin cells in the gut and actively taken up by platelets, which store most of the body 5-HT^2^,^16^. However, gut-derived 5-HT is not associated with diet-induced obesity^17^, suggesting more localized effects of 5-HT in regulating energy homeostasis. It has been known that 5-HT is present in WAT and BAT, and it promotes adipogenesis in 3T3-L1 preadipocytes^15^,^18^,^19^. Thus, we tested 5-HT production in adipose tissues. Indeed, all the genes involved in 5-HT metabolism, except for *Tph2*, were expressed in adipose tissues (Supplementary Fig. 2a). Interestingly, the HFD feeding increased the *Tph1* messenger RNA level in epididymal WAT (eWAT) and inguinal WAT (iWAT), and increased tissue 5-HT levels accordingly (Fig. 2a,b). These data suggested the potential role of adipocyte-derived 5-HT in the development of diet-induced obesity. Therefore, we investigated metabolic changes in adipose tissue.

To examine the mechanism of increased energy expenditure by PCPA treatment, we measured the metabolic activity of mouse organ by assessing ^18^fluorodeoxyglucose uptake, using positron emission tomography (PET)-computed tomography^20^. We found that the inhibition of 5-HT synthesis by PCPA significantly increased glucose uptake into BAT (Fig. 2c). Histological analysis also revealed that PCPA-treated mice showed decreased lipid droplet size and increased multilocular adipocytes in BAT (Fig. 2d). PCPA treatment increased the mRNA expression of thermogenic genes in BAT and the highest increase was observed in the *Dio2* mRNA level (Fig. 2e and Supplementary Fig. 2b). In addition, the number and size of the mitochondria and the density of the cristae were increased in the BAT of PCPA-treated mice (Supplementary Fig. 2c). These data suggested that inhibition of 5-HT synthesis increased the thermogenic activity of BAT.

**Figure 3:**
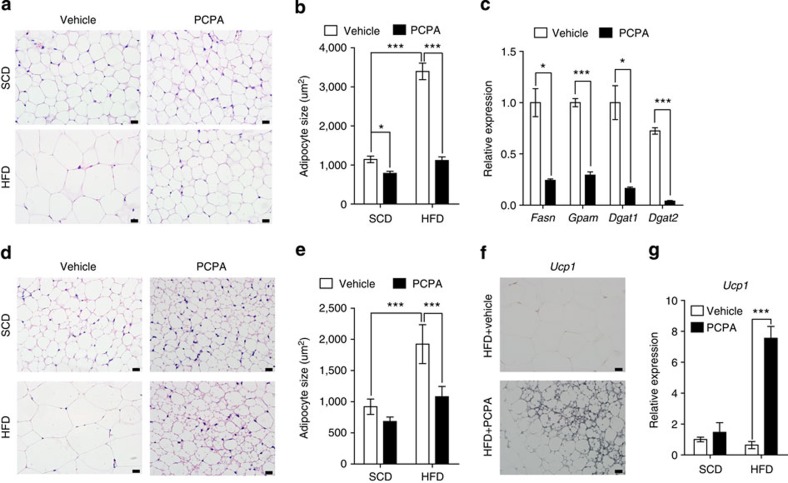
PCPA decreased lipogenesis in eWAT and induces brown fat-like changes in iWAT. (**a**) Representative haematoxylin and eosin (H&E) images of eWAT from vehicle- or PCPA-treated mice after 8 weeks of HFD feeding. (**b**) Average adipocyte sizes of eWAT were measured from H&E images using ImageJ software. *n*=5 mice per group. *P<0.05 and versus vehicle by Student's *t*-test. ***P<0.001 versus SCD+vehicle and HFD+PCPA by two-way analysis of variance (ANOVA). (**c**) Expression of genes associated with lipogenesis in eWAT, as assessed by quantitative reverse transcriptase–PCR (qRT–PCR). *n*=4 mice per group. *P<0.05 and ***P<0.001 versus vehicle by Student's *t*-test. (**d**) Representative H&E images of iWAT from vehicle- or PCPA-treated mice after 8 weeks of HFD feeding. (**e**) Average adipocyte sizes of iWAT were measured from H&E images using ImageJ software. *n*=5 mice per group. ***P<0.001 versus SCD+vehicle and HFD+PCPA by two-way ANOVA. (**f**) Immunohistochemical staining for Ucp1 in iWAT from vehicle- or PCPA-treated mice after 8 weeks of HFD feeding. (**g**) *Ucp1* gene expression in iWAT was assessed via qRT–PCR. *n*=4 mice per group. ***P<0.001 versus vehicle by Student's *t*-test. All data are presented as the mean±s.e. Scale bar, 20 μm.

In eWAT, PCPA administration led to a decrease in adipocyte size with normal cellular structures (Fig. 3a,b). The expression of lipogenic genes was decreased in PCPA-treated mice compared with control mice (Fig. 3c and Supplementary Fig. 3a). In the iWAT of PCPA-treated mice, the adipocyte size was decreased and brown adipocyte-like cells expressing Ucp1 were observed (Fig. 3d-f), indicating the browning of iWAT^21^. In agreement with the Ucp1 immunostaining, *Ucp1* and *Dio2* mRNA levels were increased in iWAT following PCPA treatment (Fig. 3g and Supplementary Fig. 3b). These results suggested that 5-HT might play a role in lipogenesis and thermogenesis in WAT.

### Peripheral Tph inhibitor prevents HFD-induced obesity

**Figure 4:**
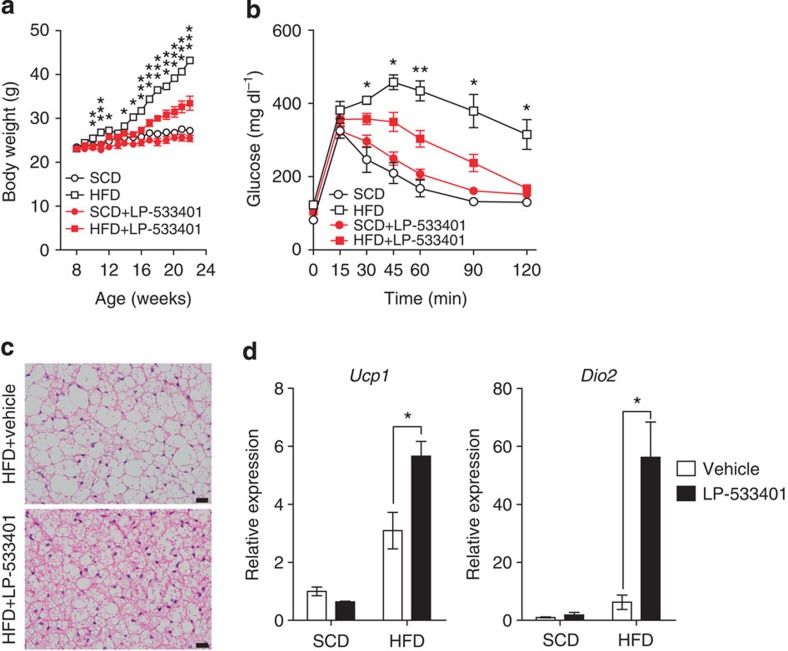
LP-533401 protects against diet-induced obesity and increased BAT activity. Mice were treated orally with vehicle or LP-533401 (30 mg kg^−1^) and fed an SCD or HFD for 14 weeks from 8 weeks of age. (**a**) Growth curves of vehicle- or LP-533401-treated mice fed an HFD. *n*=5 mice per group. *P<0.05, **P<0.01 and ***P<0.001 versus HFD+LP-533401 by Student's *t*-test. (**b**) Intraperitoneal glucose tolerance test (IPGTT) after 16 h fasting *n*=4 mice per group. *P<0.05 and **P<0.01 versus HFD+LP-533401 by Student's *t*-test. (**c**) Representative haematoxylin and eosin (H&E) images of BAT of vehicle- or LP-533401-treated mice. Scale bar, 20 μm. (**d**) Expression of thermogenesis-related genes in BAT was assessed by quantitative reverse transcriptase–PCR (qRT–PCR). *n*=4 mice per group. *P<0.05 versus vehicle by Student's *t*-test. All data are presented as the mean±s.e.

To exclude the possibility that anti-obesity effects of PCPA might be related to the inhibition of 5-HT synthesis in the brain, we have tested peripheral Tph inhibitior, LP-533401, which cannot cross the blood–brain barrier^14^. Mice treated with LP-533401 showed reduced weight gain and improved glucose tolerance compared with control mice under an HFD (Fig. 4a,b). The BAT of LP-533401-treated mice displayed similar histological changes to those observed in PCPA-treated mice after an HFD feeding (Fig. 4c). The BAT of LP-533401-treated mice also showed increased thermogenic gene expressions (Fig. 4d). Taken together, our data suggested that the inhibition of peripheral Tph1 increased energy expenditure by increasing thermogenic activity of BAT and iWAT.

### Cell autonomous function of 5-HT in adipose tissues

**Figure 5:**
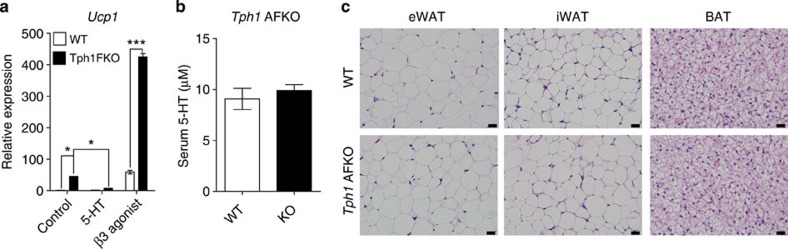
Cell autonomous function of 5-HT in adipocytes. (**a**) *Ucp1* mRNA levels were analysed by quantitative reverse transcriptase–PCR (qRT–PCR) using SVF from the BAT of *Tph1* FKO mice or their WT littermates. Cells were cultured in BAT differentiation medium for 8 days and then treated with 5-HT (10 μM) or β3AR agonist (CL 316243, 100 nM). *n*=3 mice per group. *P<0.05 for *Tph1* FKO versus WT control and Tph1 FKO-5-HT by two-way analysis of variance (ANOVA). ***P<0.001 versus WT by Student's *t*-test. (**b**) Serum 5-HT level of WT and *Tph1* AFKO mice after 6 weeks of HFD feeding. *n*=4 mice per group. (**c**) Representative haematoxylin and eosin (H&E) images of eWAT, iWAT and BAT from *Tph1* AFKO mice and their WT littermates fed an SCD. Data are presented as the mean±s.e. Scale bar, 20 μm.

To test the cell autonomous function of 5-HT in adipose tissues, we isolated the stromal vascular fraction (SVF) from BAT of adipocyte-specific *Tph1* KO (*Adipoq-Cre*^+/−^*/Tph1*^*floxlflox*^, *Tph1* FKO) mice and differentiated into mature brown adipocytes. After 8 days of culture in differentiation medium, *Ucp1* expression was upregulated in *Tph1*-null brown adipocytes, which was abrogated by 5-HT treatment (Fig. 5a). Furthermore, the increase in *Ucp1* mRNA expression by β3 adrenergic receptor (β3AR) stimulation was significantly augmented in the *Tph1*-null brown adipocytes (Fig. 5a). These data demonstrated the inhibitory role of 5-HT in thermogenic activity of BAT^22^.

**Figure 6:**
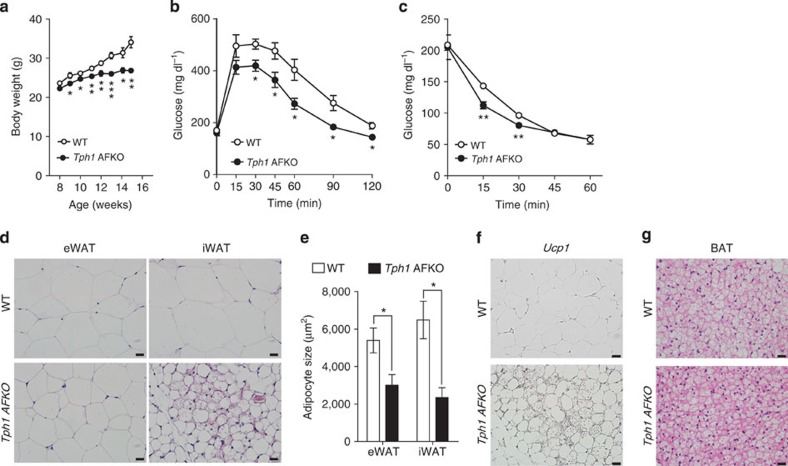
*Tph1* AFKO mice are resistant to diet-induced obesity. (**a**) Growth curves of Tph1 AFKO mice and their WT littermates fed an HFD. *n*=4 mice per group. *P<0.05, **P<0.01 and ***P<0.001 versus WT by Student's *t*-test. (**b**) Intraperitoneal glucose tolerance test (IPGTT) in HFD-fed *Tph1* AFKO mice and their WT littermates after 16h fasting. *n*=4 mice per group. *P<0.05 versus WT by Student's *t*-test. (**c**) Intraperitoneal insulin tolerance test (IPITT) in HFD-fed *Tph1* AFKO mice and their WT littermates after 4h fasting. *n*=4 mice per group. **P<0.01 versus WT by Student's *t*-test. (**d**) Representative hematoxylin and eosin (H&E) images of WAT of *Tph1* AFKO mice and their WT littermates after 6 weeks of HFD feeding. (**e**) Average adipocyte sizes of eWAT and iWAT were measured from H&E images using ImageJ software. *n*=4 mice per group. *P<0.05 versus WT by Student's *t*-test. (**f**) Immunohistochemical staining for Ucp1 in iWAT from *Tph1* AFKO mice and their WT littermates after 6 weeks of HFD feeding. (**g**) Representative H&E images of BAT of *Tph1* AFKO mice and their WT littermates after 6 weeks of HFD feeding. All data are presented as the mean±s.e. Scale bar, 20μm.

To investigate the role of 5-HT in mature adipocytes, we generated inducible *Tph1* KO (*aP2-CreERT2*^+/−^*/Tph1*^*floxlflox*^, *Tph1* AFKO) mice and induced *Tph1* KO in adipose tissues at 6 weeks of age by injecting tamoxifen intraperitoneally. *Tph1* AFKO mice looked grossly normal, maintained normal serum 5-HT levels (Fig. 5b) and no histological difference was observed in their adipose tissues (Fig. 5c). However, *Tph1* AFKO mice showed reduced weight gain, improved glucose tolerance and insulin sensitivity compared with wild-type (WT) littermates after 6 weeks of HFD feeding (Fig. 6a–c). HFD-fed *Tph1* AFKO mice showed similar histological changes in adipose tissues as the PCPA-treated mice, reduced adipocyte size in both eWAT and iWAT (Fig. 6d,e), increased *Ucp1* expression in iWAT (Fig. 6f) and increased multilocular adipocytes in BAT (Fig. 6g). These phenotypes of *Tph1* AFKO mice suggested the important role of adipocyte-derived 5-HT in the regulation of systemic energy homeostasis.

### 5-HT regulates thermogenesis via Htr3

As the anti-obesity effects of PCPA were attributed to the potentiation of adaptive thermogenesis in BAT, we attempted to identify the Htr responsible for the activation of BAT. Among Htrs in BAT (Supplementary Fig. 2a), we focused on Htr3, which is a heteropentamer of Htr3a and Htr3b, and acts as a functional 5-HT-gated cation channel^23^,^24^. Previously, we reported that Htr3 regulates glucose-stimulated insulin secretion in pancreatic islets^25^. While we were studying the effects of Htr3 in insulin secretion, we noticed that HFD-fed *Htr3a* KO (*Htr3a*^−/−^) mice showed improved insulin sensitivity and reduced weight gain after 10 weeks of age^25^,^26^. These findings prompted us to test the role of Htr3 in adaptive thermogenesis.

**Figure 7:**
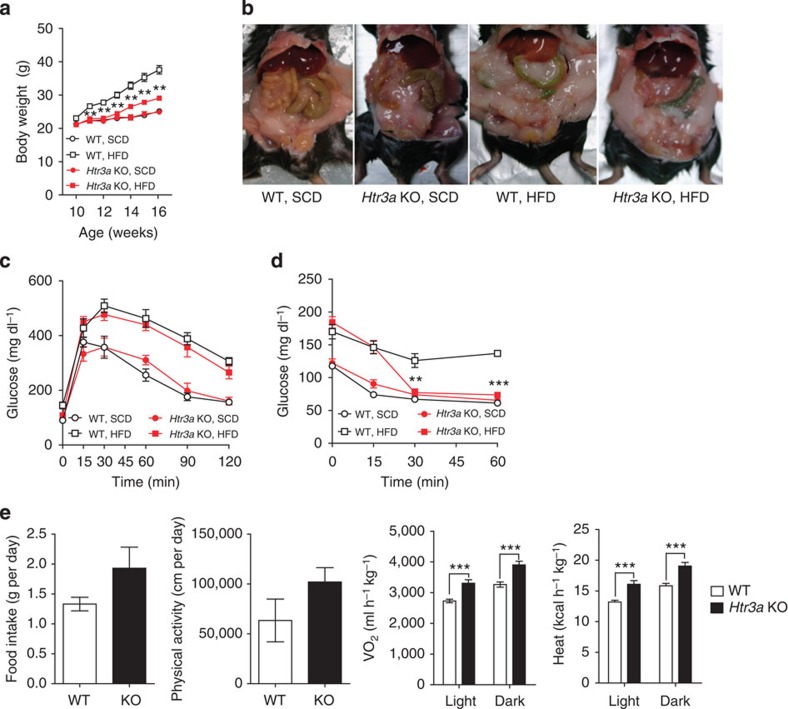
*Htr3a* KO mice are resistant to diet-induced obesity. (**a**) Growth curves of *Htr3a* KO mice and their WT littermates fed an SCD or HFD. *n*=4 mice per group. **P<0.01 versus WT, HFD by Student's *t*-test. (**b**) Gross images of *Htr3a* KO mice fed an SCD or HFD for 6 weeks beginning at 8 weeks of age. (**c**) Intraperitoneal glucose tolerance test (IPGTT) after 16 h fasting. *n*=4 mice per group. (**d**) Intraperitoneal insulin tolerance test (IPITT) after 4 h fasting. *n*=4 mice per group. **P<0.01 and ***P<0.001 versus WT, HFD by Student's *t*-test. (**e**) The metabolic rates of *Htr3a* KO mice and their WT littermates after 6 weeks of HFD feeding. *n*=4 mice per group. ***P<0.001 versus WT by Student's *t*-test. All data are presented as the mean±s.e.

**Figure 8:**
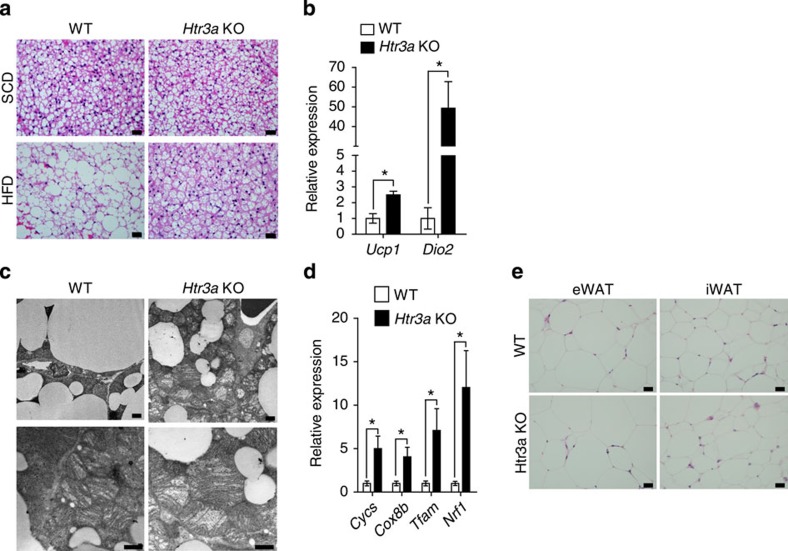
*Htr3a* KO mice shows increased BAT activity. (**a**) Representative haematoxylin and eosin (H&E) images of BAT of *Htr3a* KO mice and their WT littermates after 6 weeks of HFD feeding. Scale bar, 20 μm. (**b**) Expression of thermogenesis-related genes in BAT was assessed by quantitative reverse transcriptase–PCR (qRT–PCR). *n*=4 per group. *P<0.05 versus WT by Student's *t*-test. (**c**) Representative transmission electron microscopic (TEM) images of mitochondria in BAT from *Htr3a* KO mice and WT littermates after 6 weeks of HFD feeding. Scale bar, 1 μm. (**d**) Mitochondrial gene expressions in BAT isolated from *Htr3a* KO mice and their WT littermates after 6 weeks of HFD feeding. The mRNA expressions of mitochondrial biogenesis-associated genes were assessed using qRT–PCR. *n*=4 per group. *P<0.05 versus WT by Student's *t*-test. (**e**) Representative H&E images of eWAT and iWAT from *Htr3a* KO mice and their WT littermates after 6 weeks of HFD feeding. Scale bar, 20 μm. All data are presented as the mean±s.e.

*Htr3a* KO mice were fed an HFD for 6 weeks from 10 weeks of age and analysed their metabolic phenotypes (Supplementary Fig. 4a). As shown in Fig. 7, *Htr3a* KO mice were resistant to HFD-induced obesity (Fig. 7a,b). However, glucose tolerance was not improved in *Htr3a* KO mice, despite the improved insulin sensitivity (Fig. 7c,d). Defective insulin secretion in *Htr3a* KO mice can explain the discrepancy between glucose tolerance and insulin sensitivity^26^. As expected, *Htr3a* KO mice exhibited increased oxygen consumption and heat production compared with their WT littermates (Fig. 7e and Supplementary Fig. 4b). HFD-fed *Htr3a* KO mice did not exhibit enlarged unilocular lipid droplets in the BAT, which were observed in the BAT of their WT littermates after HFD feeding (Fig. 8a). Thermogenic gene expressions also increased in BAT of *Htr3a* KO mice (Fig. 8b). Furthermore, mitochondrial biogenesis was increased in BAT of *Htr3a* KO mice (Fig. 8c,d). These data suggested that the metabolic and histological changes observed in HFD-fed *Tph1* AFKO mice could be attributed to the reduced Htr3 activity in BAT. However, WAT of *Htr3a* KO mice did not exhibit similar changes to *Tph1* AFKO after HFD, suggesting the more selective effects of Htr3 in BAT (Figs 7b and 8e).

**Figure 9:**
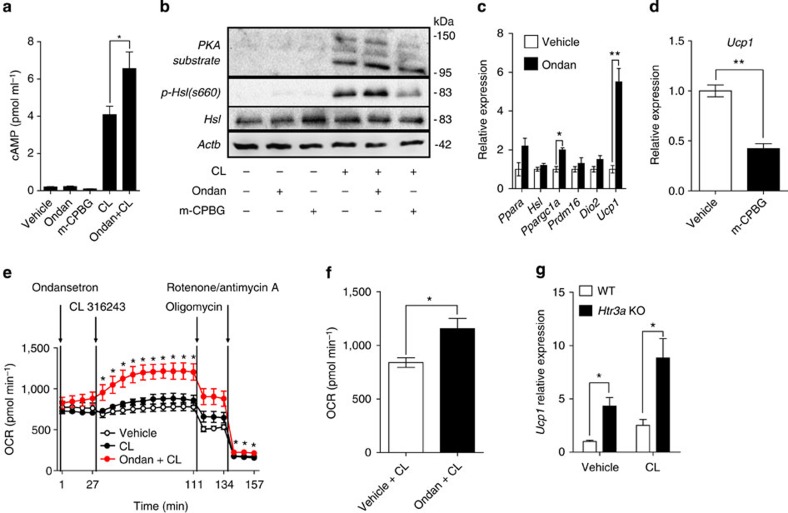
Cell autonomous effects of Htr3 on BAT activity. The effects of the Htr3 agonist and antagonist, and β3AR agonist were tested in IBA and in primary BAT from *Htr3a KO* mice. (**a**,**b**) IBA pretreated with Ondansetron (1 μM) or m-CPBG (100 nM) were treated with vehicle or CL 316243 (1 μM) for 15 min. (**a**) Intracellular cAMP levels of IBA were measured by enzyme-linked immunosorbent assay (ELISA). (**b**) Protein kinase A (PKA) substrate, *Hsl*, and phosphorylation of *Hsl* on ser660 were assessed by western blot analysis. (**c**) Expression of thermogenesis-related genes in IBA was assessed by quantitative reverse transcriptase–PCR (qRT–PCR) after 2 h ondansetron (1 μM) treatment. *n*=4 per group. *P<0.05 and **P<0.01 versus vehicle by Student's *t*-test. (**d**) *Ucp1* mRNA expression in IBA was assessed by qRT–PCR after 2 h m-CBPG (100 nM) treatment. *n*=4 per group. **P<0.01 versus vehicle by Student's *t*-test. (**e**) Oxygen consumption rate (OCR) of IBA was assessed using the Seahorse XF24 analyser. *n*=5 per group. *P<0.05 versus vehicle+CL 316243 by Student's *t*-test. (**f**) Average OCR of IBA after CL 316243 treatment. *n*=5 per group. *P<0.05 versus vehicle+CL 316243 by Student's *t*-test. (**g**) *Ucp1* mRNA expression of primary brown fat adipocytes isolated from *Htr3a* KO mice and WT littermates. *n*=4 per group. *P<0.05 versus WT by Student's *t*-test. All data are presented as the mean±s.e. Ondan, ondansetron; CL, CL 316243; m-CBPG, 1-(m-chlorophenyl)-biguanide.

To know the localized actions of Htr3 in BAT, we explored the effects of an Htr3 antagonist on immortalized brown adipocytes (IBA). After differentiation, cells were treated with an Htr3 antagonist (ondansetron) or an Htr3 agonist (1-(m-chlorophenyl)-biguanide, m-CPBG), and thermogenic response to β3AR stimulation was tested. Ondansetron and m-CPBG did not have significant effects on IBA in the absence of the β3AR agonist (Fig. 9a,b). However, ondansetron increased cyclic AMP production and phosphorylation of hormone-sensitive lipase (Hsl) and protein kinase A substrate in the presence of the β3AR agonist (Fig. 9a,b and Supplementary Fig. 4c). Ondansetron also increased the mRNA expression of thermogenic genes, such as *Ucp1* and *Ppargc1a*, in IBA (Fig. 9c). Conversely, m-CPBG decreased *Ucp1* mRNA in IBA (Fig. 9d). To examine whether blocking of Htr3 increases the energy metabolism of brown adipocytes, we measured the oxygen consumption rate (OCR) of IBA using XF analyser. Ondansetron increased the OCR synergistically with the β3AR agonist (Fig. 9e,f). *Ex vivo* experiment using primary BAT from *Htr3a* KO mice showed similar results. Primary brown adipocytes lacking Htr3 showed higher *Ucp1* expression and higher sensitivity to β3AR stimulation (Fig. 9g). Taken together, these data indicated that 5-HT regulates thermogenesis in BAT through Htr3 in cell autonomous manner.

### 5-HT regulates lipogenesis via Htr2a

**Figure 10:**
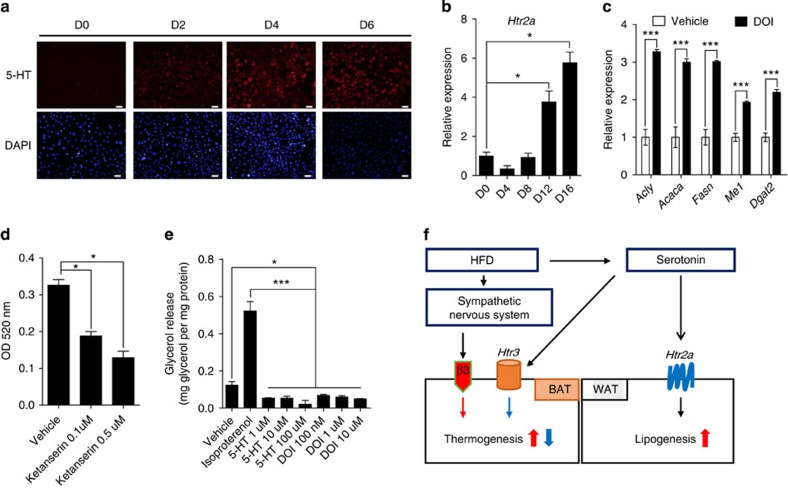
Htr2a regulates lipogenesis in WAT. (**a**) The immunofluorescence staining of 5-HT in 3T3-L1 adipocytes during differentiation. Scale bar, 20 μm. (**b**) *Htr2a* mRNA expression in 3T3-L1 adipocytes during differentiation was assessed by quantitative reverse transcriptase–PCR (qRT-PCR). *n*=3 per group. *P<0.05 versus D0 by one-way analysis of variance (ANOVA). (**c**) Lipogenic gene expression in differentiated 3T3-L1 adipocytes was assessed by qRT–PCR after Htr2a agonist (100 nM DOI) treatment for 24 h. *n*=3 per group. ***P<0.001 versus vehicle by Student's *t*-test. (**d**) Lipid accumulation in 3T3-L1 adipocytes was quantified. Cells were stained with Oil Red O and optical density (OD) of cell extracts was quantified using a spectrophotometer. *n*=4 per group. *P<0.05 versus vehicle by one-way ANOVA. (**e**) Lipolysis was assessed by a glycerol release assay in differentiated 3T3-L1 adipocytes. *n*=6 per group. *P<0.05 versus vehicle and ***P<0.001 versus isoproterenol by one-way ANOVA. (**f**) A proposed model for the regulation of energy homeostasis by 5-HT in adipocytes. Red arrows denote activation and blue arrows denote suppression. All data are presented as the mean±s.e. Vehicle: PBS; DOI (2,5-dimethoxy-4-iodoamphetamine): Htr2a agonist; ketanserin: Htr2a antagonist.

In contrast to the results of Tph1 inhibition genetically or pharmacologically, *Htr3a* KO mice maintained substantial amount of eWAT mass and no histological differences were observed in the eWAT and iWAT compared with the WT littermates. These results suggested that the effect of Htr3 inhibition is more selective in BAT, and that another mechanism could be responsible for the lipogenesis of WAT. In an attempt to identify the additional mechanism that could explain the reduction of eWAT mass caused by inhibiting Tph1 we focused on Htr2a, because recent genetic association studies have reported that *HTR2A* is significantly associated with obesity^27^,^28^,^29^. In addition, 5-HT is an adipogenic inducer of 3T3-L1 adipocytes and *Htr2a* expression is increased in hypertrophied 3T3-L1 adipocytes^15^,^18^. Indeed, 3T3-L1 adipocytes synthesized 5-HT during differentiation (Fig. 10a) and the expression of *Htr2a* gradually increased after day 8 (Fig. 10b). These results suggest that 5-HT may regulate lipogenesis in mature adipocytes through Htr2a. Therefore, we treated 3T3-L1 adipocytes with an Htr2a agonist (2,5-dimethoxy-4-iodoamphetamine, DOI) or an Htr2a antagonist (ketanserin), and assessed mRNA expression of lipogenic genes. DOI increased the mRNA levels of lipogenic genes in mature adipocytes (Fig. 10c). On the other hand, ketanserin decreased lipid accumulation (Fig. 10d). In the glycerol release assay, 5-HT and the DOI suppressed lipolysis in mature adipocytes (Fig. 10e). These data indicated that 5-HT positively regulates lipogenesis in mature adipocytes through Htr2a.

## Discussion

Although most studies on the effects of 5-HT on obesity have been focused on its central action^5^,^6^,^7^,^8^, recent studies have reported the relationship between peripheral 5-HT and obesity^30^,^31^. Genetic studies have reported that polymorphisms in human Htr genes (for example, *HTR1A*, *HTR1Dβ* and *HTR2A*) are associated with obesity^32^,^33^,^34^. A recent study using *Tph1* KO mice reported that inhibition of peripheral Tph1 protects against diet-induced obesity and promotes BAT thermogenesis^35^. They found that inhibition of Tph1 increases the sensitivity of BAT to β3AR stimulation and its effects depend on Ucp1-mediated thermogenesis.

Most peripheral 5-HT is produced in enterochromaffin cells in the gut and stored in platelets. However, gut-specific *Tph1* KO mice did not show resistance to diet-induced obesity^17^, which led us to focus on adipose tissue-derived 5-HT. In the present study, we demonstrated that adipocytes can produce 5-HT separately from the gut and HFD increases *Tph1* mRNA expression and tissue 5-HT levels in adipose tissues. Mice with inducible *Tph1* KO in adipose tissues were resistant to HFD-induced weight gain and their glycemic control was improved.

Adaptive thermogenesis was enhanced in BAT of *Htr3a* KO mice after HFD feeding, which indicated that 5-HT regulates thermogenesis of BAT via Htr3. Previously, we reported that Htr3 activation depolarizes the β-cell membrane, thereby increasing glucose-stimulated insulin secretion in pancreatic islets^25^. The membrane potential is also important in BAT activity. The activation of BAT in response to β3AR stimulation involves a transient hyperpolarization of membrane potential^36^, suggesting the possibility that Htr3 inhibition could enhance the responsiveness of BAT to β3AR stimulation through membrane hyperpolarization.

Although *Htr3a* KO mice showed reduced weight gain in HFD-induced obesity model, their WAT did not show remarkable differences in fat mass and histology compared with WAT of WT littermates (Figs 7a,b and 8e), suggesting that insulin resistance of *Htr3a*-null WAT was comparable to WT WAT. Thus, the improved insulin sensitivity of *Htr3a* KO mice is probably due to the enhanced adaptive thermogenesis in BAT. In this context, obese WAT of *Htr3a* KO mouse could be considered as a neutral bystander, otherwise insulin sensitivity would not be improved in HFD-fed *Htr3a* KO mice. In addition, the inhibition of 5-HT synthesis under HFD resulted in the decreased lipogenesis in WAT and the increased thermogenesis in BAT, suggesting the role of different Htr in WAT. Indeed, *in vitro* experiments using 3T3-L1 adipocytes showed that Htr2a agonist treatment increased lipid accumulation and 5-HT suppressed lipolysis. Taken together, these results suggested that 5-HT could regulate energy storage in WAT through Htr2a and energy expenditure in BAT through Htr3.

In the present study, we provide evidence for a complex model, explaining the regulation of energy metabolism in different adipose tissues (Fig. 10f). In the over-fed state, 5-HT level increased in WAT, leading to the augmentation of lipogenesis via Htr2a. 5-HT also suppressed thermogenesis in the BAT via Htr3. When 5-HT signalling was inhibited, lipogenesis decreased in the eWAT and thermogenesis increased in both iWAT and BAT. The β3AR signalling stimulated by an HFD coupled with uninhibited thermogenesis by the blocking of Htr3 signalling resulted in enhanced energy expenditure in BAT. Thus, the inhibition of 5-HT production in adipose tissues may represent a novel strategy for anti-obesity treatment.

## Methods

### Reagents

PCPA, D-glucose, insulin, CL 316243, ondansetron, m-CPBG, triiodothyronine (T3), 3-isobutyl-1-methylxanthine (IBMX), indomethacin, dexamethasone, Oil Red O dye, ketanserin, DOI, isopropyl alcohol (IPA), formalin, ascorbic acid, perchloric acid, tamoxifen and polyethylene glycol 400 (PEG-400) were purchased from Sigma (St Louis, MO, USA). LP-533401 was purchased from Dalton Pharma Services (Toronto, Ontario, Canada). TRIzol reagent, DMEM medium, calf serum, fetal bovine serum (FBS) and penicillin/streptomycin (P/S) were obtained from Invitrogen (Carlsbad, CA, USA).

### Animals and diets

The generation of *Tph1*^*flox/flox*^ mice, Adipoq-Cre mice and aP2-CreERT2 mice has previously been reported^37^,^38^,^39^. C57BL/6 J mice, *Htr3a* KO mice (B6.129X1-*Htr3a*^*tm1jul/J*^) and ob/ob mice (B6.V-*Lep*^*ob*^/J) were purchased from the Jackson Laboratory (Bar Harbor, ME, USA). To generate the adipocyte-specific *Tph1* KO (*Tph1* FKO) mice and inducible *Tph1* KO (*Tph1* AFKO) mice, *Tph1*^*flox/flox*^ mice were crossed with Adipoq-Cre mice and aP2-CreERT2 mice, respectively. The mice were housed in climate-controlled, specific pathogen-free barrier facilities under a 12-h light–dark cycle, and chow and water were provided *ad libitum*. The Institutional Animal Care and Use Committee at the Korea Advanced Institute of Science and Technology approved the experimental protocols for this study. *Htr3a* KO mice and *Tph1*^*flox/flox*^ mice were backcrossed with C57BL/6 J mice for more than ten generations. Cre recombination of 6-week-old *Tph1* AFKO mice was induced by intraperitoneal injection of five doses of 2 mg of tamoxifen (Sigma) for a week. Male mice (aged 8∼10 weeks) were fed either an SCD (12% fat calories, Purina Laboratory Rodent Diets 38057) or an HFD (60% fat calories, Research Diets D12492). PBS or 300 mg kg^−1^ PCPA was administered as a daily intraperitoneal injection. LP-533401 was dissolved in PEG-400 and 5% dextrose (40:60 ratio). Vehicle or 30 mg kg^−1^ LP-533401 was administered daily with a feeding needle. We randomly divided C57BL/6J mice into two to approximately four groups. For transgenic mice, we compared data between KO mice and their WT littermates. No blinding was performed.

### Cell culture

Murine 3T3-L1 cells (American Type Culture Collection) were cultured in DMEM supplemented with 10% FCS and 100 μg ml^−1^ P/S in a humidified atmosphere of 5% CO_2_ at 37 °C. Two days after reaching confluence, the cells were induced to differentiate using medium supplemented with 0.5 mM IBMX, 1 mg ml^−1^ insulin and 1 μM dexamethasone (day 0). After 2 days, the medium was replaced with DMEM supplemented with 1 mg ml^−1^ insulin in 10% FBS and P/S (day 2), and it was changed every 2 days from day 4 to 8. IBA were cultured in DMEM supplemented with 10% FBS and P/S in a humidified atmosphere of 5% CO_2_ at 37 °C^40^. After reaching 95% confluence, the cells were induced to differentiate using DMEM with 10% FBS, 0.5 μg ml^−1^ insulin, 1 nM T3, 0.125 mM indomethacin, 2 μg ml^−1^ dexamethasone, 0.5 μM IBMX and P/S (day 0). After 2 days, the medium was replaced with DMEM supplemented with 10% FBS, 0.5 μg ml^−1^ insulin, 1 nM T3 and P/S (day 2), and it was changed every 2 days from day 4 to 8. Primary brown pre-adipocytes were prepared from newborn *Htr3*a KO mice and their WT littermates as described previously^41^. The interscapular BAT was isolated from mice at postnatal day 2∼3, minced and then incubated in isolation buffer (0.123 M NaCl, 5 mM KCl, 1.3 mM CaCl_2_, 5 mM glucose, 100 mM HEPES and 4% BSA) containing 1 mg ml^−1^ collagenase type II (LS004176, Worthington) in 37 °C shaking incubator for 45∼50 min. The digested tissue was filtered through a 100-μm mesh filter (BD Bioscience). These filtered cells were washed twice with culture media and these pre-adipocytes were then cultured in DMEM supplemented with 20% FBS and 100 μg ml^−1^ P/S in a humidified atmosphere of 5% CO_2_ at 37 °C. After reaching 95% confluence, the cells were induced to differentiate using DMEM with 20% FBS, 20 nM insulin, 1 nM T3, 0.125 mM indomethacin, 0.5 mM dexamethasone, 0.5 mM IBMX and P/S (day 0). After 2 days, these cells were maintained in DMEM supplemented with 20% FBS, 20 nM insulin, 1 nM T3 and P/S for 4–5 days until exhibiting a massive accumulation of fat droplets^41^. We confirmed that these cell lines were free from *Mycoplasma* infection.

### SVF isolation

The SVF of BAT from 7-week-old mice was separated by collagenase digestion. Briefly, the adipose tissues were dissected, minced and digested with 0.2% collagenase A (Roche) in Hank's balanced salt solution (Sigma) for 45 min at 37 °C, with constant shaking. Mature adipocytes and connective tissues were separated from the cell pellet by centrifugation at 800*g* for 10 min at 4 °C. The cell pellet was then suspended with RBC lysis buffer (Sigma) and filtered through a 40-μm mesh filter (BD Bioscience). The pelleted stromal vascular cells were re-suspended in DMEM containing 10% FBS and seeded in six-well plates for adipogenic differentiation.

### Oil Red O staining

After full differentiation (day 8), 3T3-L1 adipocytes were fixed with 3.7% (w/v) formaldehyde in PBS for 15 min at room temperature and then washed three times with PBS. The cells were then stained with filtered Oil Red O solution (1.5 mg ml^−1^ 60% (v/v) IPA) for 30 min and rinsed twice with distilled water. To quantify the amount of Oil Red O staining, the cells were eluted with 100% IPA for 10 min and the absorbance densities of the extracts were measured at 520 nm, using a VersaMax microplate reader (Molecular Devices, Sunnyvale, CA, USA).

### Metabolic analysis

To measure the metabolic rate, the mice were housed individually in an eight-chamber, open-circuit Oxymax/CLAMS (Columbus Instruments Comprehensive Lab Animal Monitoring System) system as previously described^42^. Each mouse was assessed for 72 h in the fed state to measure metabolic rates. The respiratory exchange ratio (RER=VCO_2_/VO_2_) and heat production (HP=(3.185+1.232 × RER) × VO_2_) were calculated^43^. PET imaging was performed using a microPET R4 scanner (Concorde Microsystems, Siemens) as previously described^44^.

### Glucose tolerance test and insulin tolerance test

For the glucose tolerance tests, the mice were administered 2 g kg^−1^ D-glucose in PBS after overnight fasting. For the insulin tolerance tests, the mice were intraperitoneally injected with insulin (0.75 U kg^−1^) after 4 h fasting. The blood samples were obtained from tail veins at 0, 15, 30, 45, 60, 90 and 120 min after injection and glucose concentrations were measured using a Gluco DR Plus glucometer (Allmedicus, Korea).

### Blood chemistry analysis

Tissue 5-HT was extracted by homogenization in extraction buffer containing 0.02% ascorbic acid in 0.1 M perchloric acid followed by centrifugation. The 5-HT levels in the supernatants were measured via the liquid chromatography–mass spectrometry method.

### cAMP assay

Differentiated IBA were treated with 1 μM ondansetron or 100 nM m-CPBG or 1 μM CL 316243 for 15 min. CL 316243 was used as a positive control. The cAMP competitive ELISA (Promega) was performed according to the manufacturer's instruction. Briefly, cAMP was extracted by adding 0.1 M HCl with 0.5% Triton X-100 to the cells. After centrifugation at 600*g* for 10 min, the supernatant was used for the determination of cAMP levels by competitive cAMP ELISA.

### Oxygen consumption rate (OCR) assay

The OCR of the cells was measured using a Seahorse XF analyser (Seahorse Bioscience, Billerica, MA, USA). After seeding IBA on an XF-24 plate, the cells were incubated and differentiated using the protocol described above. Fully differentiated IBA were pre-treated with PBS and ondansetron for 30 min. The IBA were then treated with the β3AR agonist and the mitochondrial inhibitors oligomycin and rotenone/antimycin. The OCRs were calculated and recorded by a sensor cartridge and the Seahorse XF-24 software.

### Quantitative reverse transcriptase-PCR (qRT-PCR) analysis

Total RNA was extracted from the mouse tissues or cell lines using TRIzol reagent, according to the manufacturer's protocol. After TURBO DNase (Invitrogen) treatment, 2 μg of total RNA was used to generate complementary DNA with Superscript III reverse transcriptase (Invitrogen). To analyse gene expression, real-time PCR was performed with a ViiA 7 Real-Time PCR system (Applied Biosystems) and the Power SYBR Green PCR master mix (Applied Biosystems). Relative quantification was based on the ddCt method and *ActB* was used as an endogenous control (internal control). The primer sequences are provided in the Supplementary Table 1.

### Histological analysis

Inguinal, epididymal and interscapular adipose tissues were harvested, fixed in 4% (w/v) paraformaldehyde in PBS and embedded in paraffin. Then, 5-μm-thick tissue sections were deparaffinized, rehydrated and used for haematoxylin and eosin staining, immunohistochemistry and immunofluorescence. For antigen retrieval, the slides were submerged in 10 mM sodium citrate (pH 6.0) and heated to 95 °C for 20 min. Visualization of Ucp1 and Plin1 was performed using a VECTASTAIN ABC Kit (PK-4001, Vector Laboratories, Burlingame, CA, USA), according to the manufacturer's instructions. Briefly, the slides were incubated with BLOXALL Blocking solution (SP-6000, Vector Laboratories) followed by incubation with 2% normal goat serum for 30 min at room temperature, to block nonspecific binding. Sections were incubated with primary antibody against Ucp1 (ab10983, Abcam) or Plin1 (ab3526, Abcam) for 1 h at room temperature, followed by 30 min incubation with a species-specific, biotinylated secondary antibody. The slides were incubated with Vectastain ABC-AP reagent for 30 min and then incubated with alkaline phosphatase substrate (DAB, SK-4100, Vector Laboratories) for visualization. The stains and antibodies used for the immunofluorescence staining included BODIPY (BODIPY493/503, Invitrogen), anti-5-HT (ab10385, Abcam) and DAPI (D9542, Sigma). Electron microscopy images of BAT were obtained by transmission electron microscopy (Tecnai Spirit TEM) as previously described^45^. Briefly, the BAT was first fixed with 2.5% glutaraldehyde, and after fixation ultra-thin sections were cut, stained with uranyl acetate and lead citrate, and then examined under an electron microscope.

### Western blot analysis

Whole-cell lysates were extracted by incubating cells in RIPA buffer (25 mM Tris-HCl pH 7.6, 150 mM NaCl, 1% NP-40, 1% sodium deoxycholate, 0.1% SDS) plus protease inhibitors (Roche). Supernatants were collected following a brief centrifugation and protein concentrations in the supernatants were measured using the BCA Protein Assay Kit (Thermo Scientific, Rockford, IL, USA). The cell lysates were then mixed with equal volumes of 2 × Laemmli buffer (4% SDS, 20% glycerol, 10% 2-mercaptoethanol, 0.01% bromophenol blue and 120 mM Tris-HCl pH 6.8) and boiled for 5 min at 95 °C. Next, the protein samples were separated by SDS–PAGE and transferred to a polyvinylidene difluoride membrane (Millipore). After blocking in a 5% skim milk solution (Sigma), the membranes were incubated with the following specific primary antibodies: anti-phospho-HSL (Ser660) antibody (diluted 1:500, Cell Signaling #4126), anti-phospho-(Ser/Thr) protein kinase A substrate antibody (diluted 1:500, Cell Signaling #9621) and anti-Act antibody (diluted 1:1000, Cell Signaling #3700). The membranes were then washed with 1 × TBST and incubated with anti-rabbit IgG horseradish peroxidase-linked antibody or anti-mouse IgG antibody. The detection of each protein was performed using Supersignal West Pico Chemiluminescent Substrate (Thermo Scientific), according to the manufacturer's instructions. Signals were captured by a ChemiDoc MP system (Bio-Rad).

### Glycerol release assay

Lipolysis was measured as the rate of glycerol release using a free glycerol reagent (Sigma), following the manufacturer's protocol. Briefly, fully differentiated 3T3-L1 adipocytes in a 24-well plate were incubated with 5-HT or DOI in Krebs Ringer phosphate buffer (136 mM NaCl, 4.7 mM KCl, 10 mM NaPO_4_, 0.9 mM MgSO_4_ and 0.9 mM CaCl_2_) containing 4% fatty acid-free BSA (Sigma) for 24 h. Isoproterenol was used as a positive control. After incubation, 10 μl of the cell culture supernatant was mixed with 0.8 ml of the free glycerol reagent and the mixture was then incubated at 37 °C for 5 min. The absorbance of the sample was determined at 540 nm using a spectrophotometer (DU730 Life Science UV/Vis, Beckman Coulter, Indianapolis, IN, USA). The amount of released glycerol was expressed relative to the cellular protein content.

### Statistics

All values are expressed as the mean and s.e.m. The groups were compared by Student's *t*-test or one-way analysis of variance (ANOVA) or two-way ANOVA. Normal distribution was tested by the *f*-test. *P*-values <0.05 were considered statistically significant.

## Author contributions

C.-M.O., J.N., I.K.L., S.P. and H.K. generated the hypothesis, designed the experiments and wrote the manuscript. M.S., V.Y., G.K., S.K., C.-M.O., J.N., Y.G., K.E.S., K.K., H.K., B.Y.B., H.W.L., Y.H.J., J.H.S., I.K.L., S.P. and H.K. performed experiments and analysed data.

## Additional information

**How to cite this article**: Oh, C.-M. *et al*. Regulation of systemic energy homeostasis by serotonin in adipose tissues. *Nat. Commun*. 6:6794 doi: 10.1038/ncomms7794 (2015).

## Supplementary Material

Supplementary Information Supplementary Figures 1-4 and Supplementary Table 1
