# Supplementary material for: Regulation of systemic energy homeostasis by serotonin in adipose tissues
Source: Nat Commun. 2015 Apr 13;6:6794. doi: 10.1038/ncomms7794 (PMC4403443; doi:10.1038/ncomms7794)
Supplement: Supplementary Information — Supplementary Figures 1-4 and Supplementary Table 1 [file ncomms7794-s1.pdf]

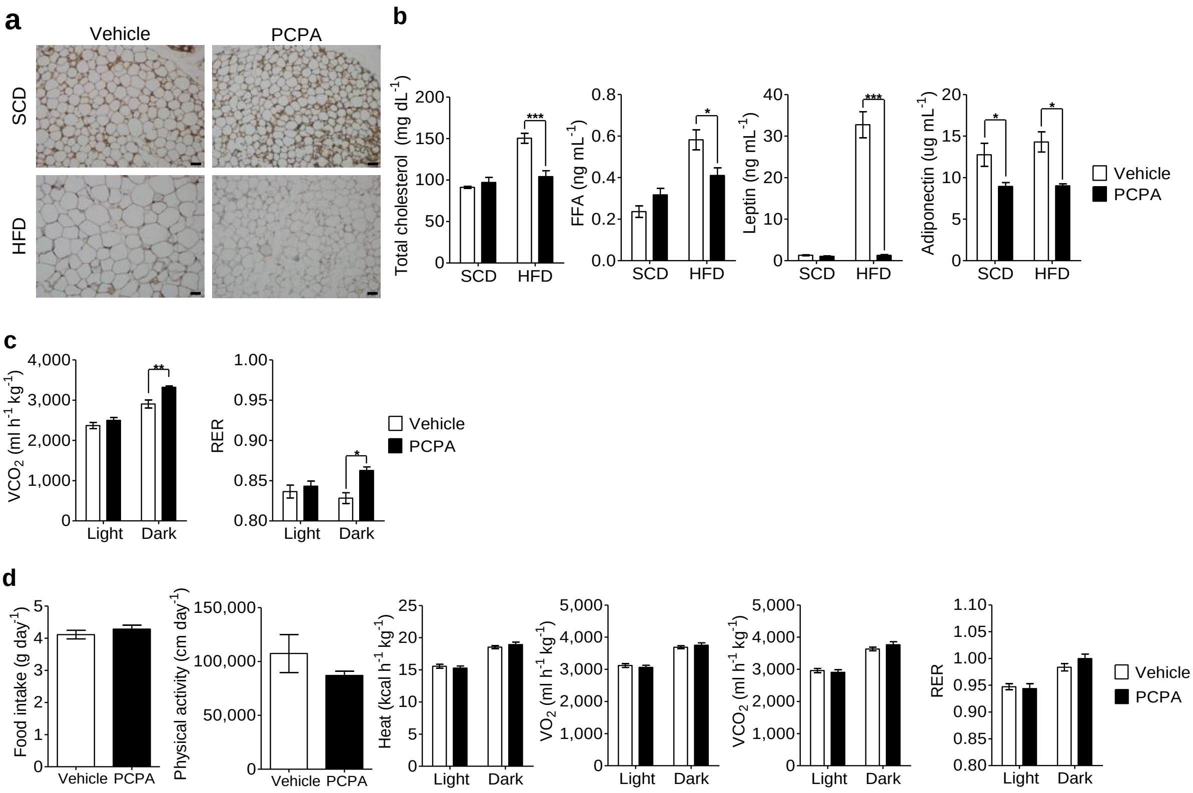

Supplementary Figure 1. Histology and metabolic profiles of PCPA-treated mice.

(**a**) Immunohistochemical staining for Plin1 of eWAT after vehicle (PBS) or PCPA treatment (300mg kg^{-1}) for 6 weeks. The scale bar indicates 20 μm. (**b**) Effects of PCPA on total cholesterol, free fatty acid, leptin and adiponectin. Serum levels of total cholesterol, FFA, leptin and adiponectin were measured by ELISA. *n*=4 mice per group. **P*<0.05 and ****P*<0.001 vs. vehicle by Student's *t*-test. (**c**) The metabolic profiles of 14-week-old vehicle- or PCPA-treated mice were measured using the Oxymax system. Mice were acclimatized for 24 hours and data were collected for 48 hours. *n*=4 mice per group. **P*<0.05 and ***P*<0.01 vs. vehicle by Student's *t*-test. (**d**) The metabolic profiles of 14-week-old SCD-fed mice were measured using indirect calorimetry after treatment with vehicle or PCPA (300mg kg^{-1}) for 6 weeks. *n*=4 mice per group. All data presented as mean ± standard error.

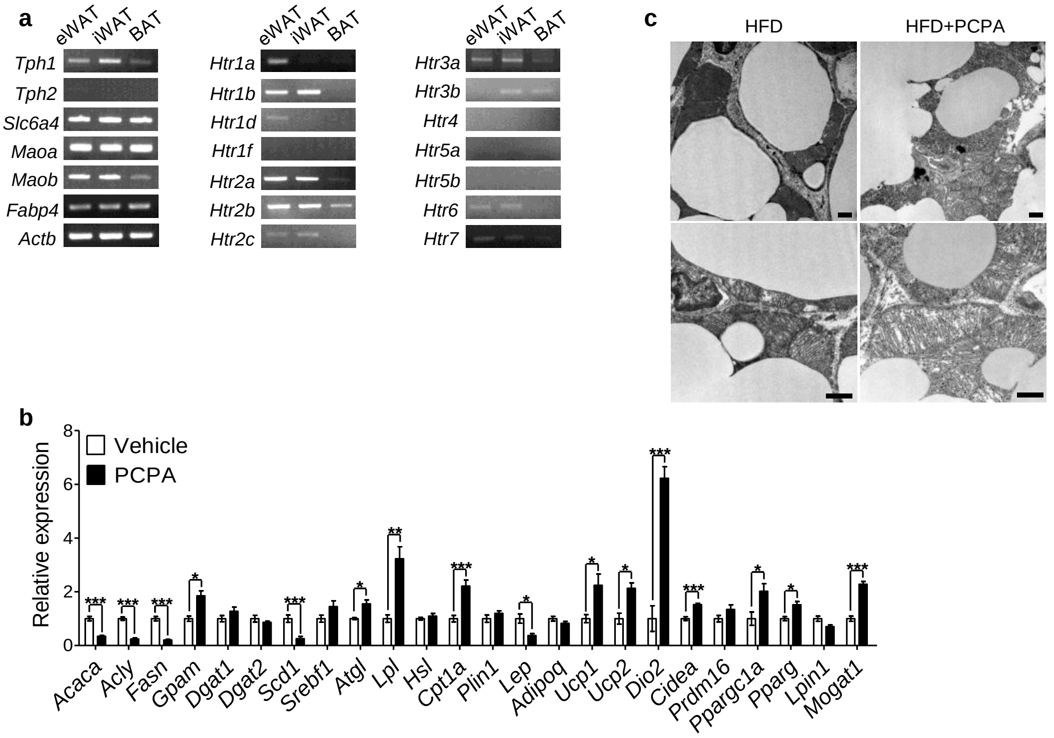

Supplementary Figure 2. 5-HT system in adipose tissue and characteristics of brown adipose tissue of PCPA-treated mice.

(**a**) The mRNA expressions of *Tph1* and Htrs in mouse adipose tissues were analyzed by RT-PCR. Adipose tissues were isolated from eWAT, iWAT and BAT of C57BL/6J mice at 8 weeks of age. (**b**) The mRNA expression of genes involved in lipid metabolism and thermogenesis. The BATs of vehicle (PBS)- or PCPA-treated mice were isolated after 6 week of HFD feeding and their mRNA levels were measured using quantitative RT-PCR (qRT-PCR). n=4 mice per group. *P<0.05, **P<0.01 and ***P<0.001 vs. vehicle by Student's t-test. Data are presented as the mean ± standard error. (**c**) Representative transmission electron microscopy images of mitochondria in PCPA-treated BAT. BAT was isolated from mice fed an HFD for 6 weeks with or without PCPA treatment. The BAT from HFD-fed mice with PCPA treatment displayed an increased size and number of mitochondria. The scale bar indicates 1 μm.

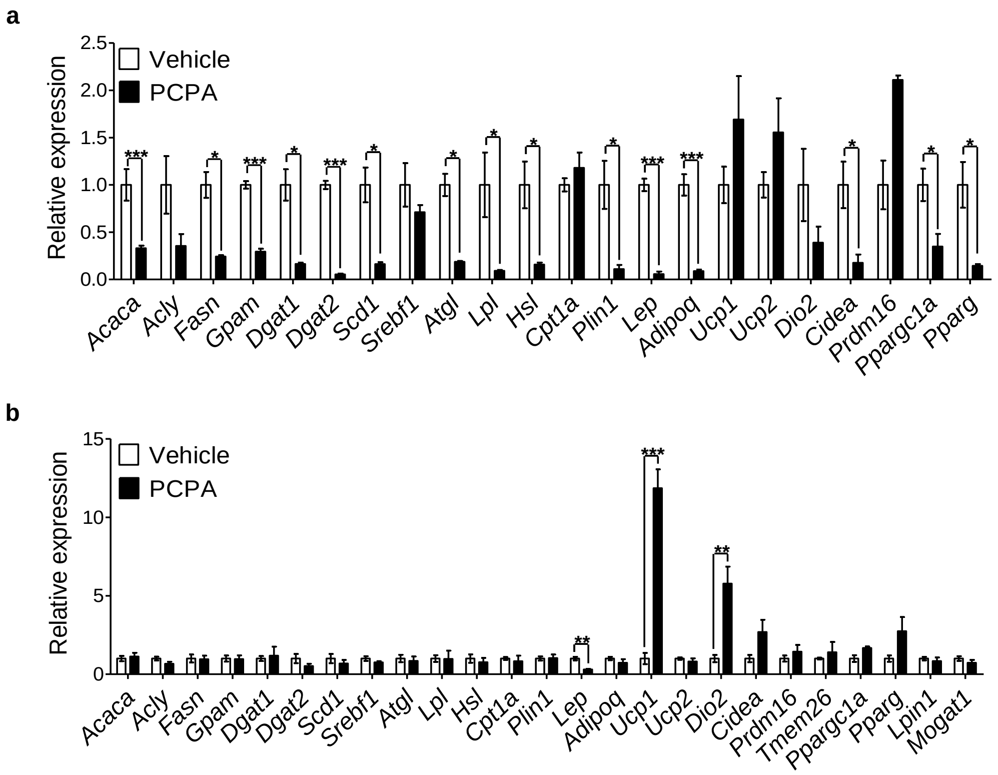

Supplementary Figure 3. Effects of 5-HT depletion on the gene expression in adipose tissues.

Mice were treated with vehicle (PBS) or PCPA (300 mg kg^{-1}) for 6 weeks beginning at 8 weeks of age. During the treatment period, these mice were fed an HFD. After 6 weeks of treatment, WATs were isolated, and the mRNA levels for the genes involved in lipid metabolism and thermogenesis were measured using qRT-PCR. Gene expressions of eWAT (**a**) and iWAT (**b**). *n*=4 mice per group. *$P<0.05$, **$P<0.01$ and ***$P<0.001$ vs. vehicle by Student's *t*-test. All data are presented as the mean ± standard error.

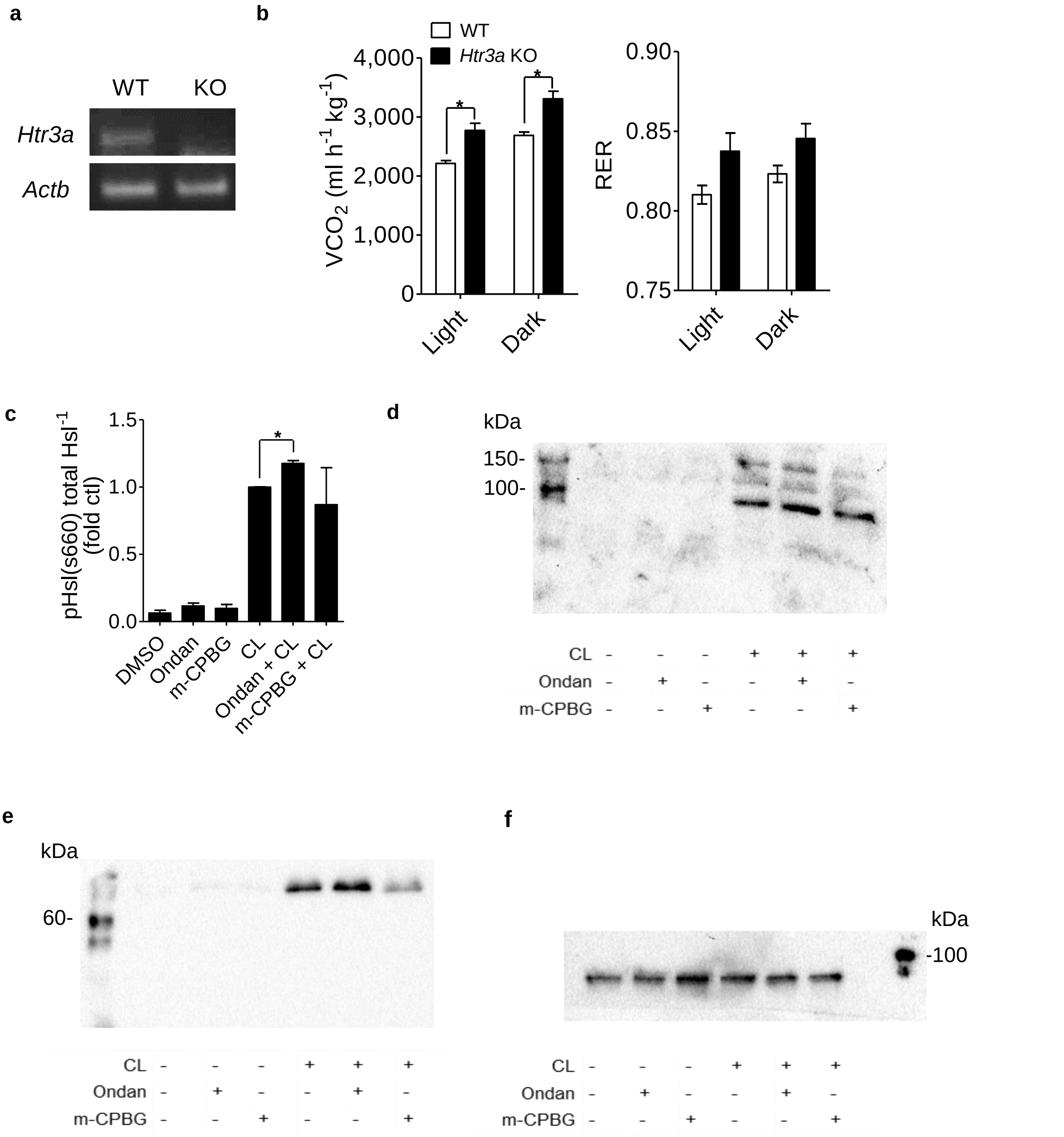

Supplementary Figure 4. Metabolic rates of *Htr3a* KO mice and phosphorylation of HSL, PKA substrate of immortalized brown adipocytes after β3AR stimulation.

(**a**) *Htr3a* mRNA expression of BAT from *Htr3a* KO mice and WT littermates at 8 weeks of age. (**b**) The metabolic profiles of 14-week-old *Htr3a* KO mice and WT littermates were

measured using the Oxymax system after fed an HFD for 6 weeks. Mice were acclimatized for 24 hours, and data were collected for 48 hours. *n*=4 mice per group. *$P<0.05$ vs. WT by Student's *t*-test. (**c**) Relative Hsl phosphorylation in immortalized brown adipocytes (IBAs). IBAs were pretreated with DMSO, Ondansetron or m-CPBG for 30 minutes. Then these IBAs were treated PBS or CL 316243 for 15 minutes. Hsl phosphorylation was analyzed by western blot assay. *n*=3 per group. *$P<0.05$ vs. CL 316243 by Student's *t*-test. (**d-f**) Western blot images with size marker of Fig. 5b: PKA substrate (**d**), *p*-HSL (s660) (**e**), HSL (**f**). All data are presented as the mean ± standard error.

Supplementary Table 1. PCR primer sequences

	Forward Primer (5'-3')	Reverse Primer (5'-3')
Acaca	CAGTAACCTGGTGAAGCTGGA	GCCAGACATGCTGGATCTCAT
Acly	CCCTCTTCAGCCGACATACC	CTGCTTGTGATCCCCAGTGA
Actb	CAGCTTCTTTGCAGCTCCTT	CTTCTCCATGTCGTCCCAGT
Adipoq	CTCCACCCAAGGGAACTTGT	GGACCAAGAAGACCTGCATC
Cidea	GCCGTGTTAAGGAATCTGCTG	TGCTCTTCTGTATCGCCCAGT
Cox8b	GAACCATGAAGCCAACGACT	GCGAAGTTCACAGTGGTTCC
Cpt1a	AGCTCGCACATTACAAGGACA	CCAGCACAAAGTTGCAGGAC
Cycs	GCAAGCATAAGACTGGACCAAA	TTGTTGGCATCTGTGTAAGAGAATC
Dgat1	GGATCTGAGGTGCCATCGTC	ATCAGCATCACCACACACCA
Dgat2	CATCATCGTGGTGGGAGGTG	TGGGAACCAGATCAGCTCCAT
Dio2	TTGGGGTAGGGAATGTTGGC	TCCGTTTCCTCTTTCCGGTG
Fabp4	AACACCGAGATTTCCTTCAA	TCACGCCTTTCATAACACAT
Fasn	AAGCGGTCTGGAAAGCTGAA	AGGCTGGGTTGATACCTCCA
Gpam	CCACAGAGCTGGGAAAGGTT	GTGCCTTGTGTGCGTTTCAT
Hsl	AACGAGACAGGCCTCAGTGT	GAATCGGCCACCGGTAAAGA
Htr1a	TCAGCTACCAAGTGATCACCTCT	GTCCACTTGTTGAGCACCTG
Htr1b	TGCTCCTCATCGCCCTCTATG	CTAGCGGCCATGAGTTTCTTCTT
Htr1d	CCTCCAACAGATCCCTGAATG	CAGAGCAATGACACAGAGATGCA
Htr1f	TGTGAGAGAGAGCTGGATTATGG	TAGTTCCTTGGTGCCTCCAGAA
Htr2a	AGCTGCAGAATGCCACCAACTAT	GGGATTGGCATGGATATACCTAC
Htr2b	AAATAAGCCACCTCAACGCCT	TCCCGAAATGTCTTATTGAAGAG
Htr2c	TTCTTAATGTCCCTAGCCATTGC	GCAATCTTCATGATGGCCTTAGT
Htr3a	AAATCAGGGCGAGTGGGAGCTG	GACACGATGATGAGGAAGACTG
Htr3b	CGTGTGGTACCGAGAGGTTT	GGATGGGCTTGTGGTTTCTA
Htr4	ATGGACAAACTTGATGCTAATGTGA	TCACCAGCACCGAAACCAGCA
Htr5a	GATTGACTTCAGTGGGCTCG	AAAGTCAGGACTAGCACTCG
Htr7	CTCGGTGTGCTTTGTCAAGA	TTGGCCATACATTTCCCATT

Lep	ACACACGCAGTCGGTATCC	GCAGCACATTTTGGGAAGGC
Lpin1	CATACAAAGGCAGCCACACG	CATACAAAGGCAGCCACACG
Maoa	GCGGTACAAGGGTCTGTTCC	CAGCCAATCCTGAGATGCCG
Maob	GGGCGGCATCTCAGGTATGG	AAGTCCTGCCTCCTACACGG
Me1	GACCCGCATCTCAACAAGGA	CAGGAGATACCTGTCGAAGTCA
Nrf1	CAGCAACCCTGATGGCACCGTGTC	GGCCTCTGATGCTTGCGTCGTCTG
Plin1	GGTGTTACAGCGTGGAGAGTA	TCTGGAAGCACTCACAGGTC
Pparg	GGTGTGATCTTAACTGCCGGA	GCCCAAACCTGATGGCATTG
Ppargc1a	GCCCAGGTACGACAGCTATG	ACGGCGCTCTTCAATTGCTT
Prdm16	AGCCCTCGCCCACAACTTGC	TGACCCCCGGCTTCCGTTCA
Scd1	AGAGTCAGGAGGGCAGGTTT	GAACTGGAGATCTCTTGGAGCA
Slc6a4	CGCAGTTCCCAGTACAAGC	CGTGAAGGAGGAGATGAGG
Srebf1	GTGGGCCTAGTCCGAAGC	CTGGAGCATGTCTTCGATGT
Tfam	AGTTCCCACGCTGGTAGTGT	GCGCACATCTCGACCC
Tmem26	ACCCTGTCATCCCACAGAG	TGTTTGGTGGAGTCCTAAGGTC
Tph1	ACCATGATTGAAGACAACAAGGAG	TCAACTGTTCTCGGCTGATG
Tph2	GCCATGCAGCCCGCAATGATGATG	CAACTGCTGTCTTGCTGCTC
Ucp1	CTTTGCCTCACTCAGGATTGG	CTTTGCCTCACTCAGGATTGG
Ucp2	GTGGTCGGAGATACCAGAGC	GAGGTTGGCTTTCAGGAGAG
